# Genome-wide identification of raffinose synthase gene family in barley (*Hordeum vulgare* L.) reveals its role in multiple abiotic stress tolerance

**DOI:** 10.3389/fpls.2026.1767123

**Published:** 2026-03-10

**Authors:** Dongdong Xu, Mengmeng Rui, Xiaoge Wang, Yongsheng Deng, Liying Gao, Fanjin Kong, Guifang Shen, Bing Duan, Zongwen Wang, Zongfu Han

**Affiliations:** 1 Institute of Industrial Crops, Shandong Academy of Agricultural Sciences, Jinan, Shandong, China; 2 National Center of Technology Innovation for Comprehensive Utilization of Saline-Alkali Land, Dongying, Shandong, China

**Keywords:** abiotic stress, barley, gene expression pattern, haplotype analysis, raffinose synthase

## Abstract

Raffinose synthase (RS) catalyzes the biosynthesis of raffinose from galactinol and sucrose, playing an important role in plant stress tolerance and seed development. However, the *RS* gene family has not been systematically characterized in barley. In this study, we performed a comprehensive genome-wide identification of *RS* gene family in barley. A total of eight *HvRS* genes were identified from the Morex V3 genome, which are distributed across four chromosomes. Phylogenetic analysis of *RS* homologs from other plants showed that the 79 RS proteins were classified into five distinct clades, revealing close evolutionary relationships among barley, wheat, and *Brachypodium distachyon*. Promoter cis-element analysis revealed an abundance of hormone- and stress-responsive elements, suggesting roles of *HvRS* genes in adaptive responses. Quantitative real-time PCR (qRT-PCR) analysis revealed that *HvRS2* and *HvRS5* were markedly upregulated in roots and/or shoots under salt, drought (simulated by polyethylene glycol, PEG), and abscisic acid (ABA) treatments, indicating their involvement in the early-phase abiotic stress response. Relative to time-matched controls, the *HvRS2* expression levels in roots increased to 9.90-fold at 24 h under salt stress and to 49.1-fold at 6 h under ABA treatment. Meanwhile, *HvRS5* expression levels in shoots reached 40.0-fold at 6 h under salt stress and to 26.6-fold at 12 h ABA treatment relative to controls. Pan-genome haplotype analysis of the two stress-responsive genes, *HvRS2* and *HvRS5*, revealed substantial nucleotide diversity, identifying 19 and 25 distinct haplotypes, respectively. Notably, the geographic distribution of haplotypes in domesticated barley exhibited a pronounced East-West divergence, suggesting that they may experience divergent selection during domestication. Collectively, this study offers a comprehensive systematic characterization of the *RS* gene family in barley, and highlights *HvRS2* and *HvRS5* as promising candidates for improving abiotic stress tolerance. These findings lay a solid foundation for subsequent functional validation and stress-resilience breeding in barley.

## 1 Introduction

Anthropogenic activities are accelerating global climate change, leading to a marked increase in the frequency and intensity of extreme weather events. These environmental perturbations intensify abiotic stresses including drought, frost, salinity, and heat stress, thereby severely undermining global agricultural productivity (Oyebamiji et al., 2024). To counteract these adverse impacts, plants have evolved a suite of sophisticated adaptive mechanisms. These encompass the activation of specific signal transduction pathways and the accumulation of compatible metabolites (Sharma et al., 2019). Among these protective compounds, raffinose family oligosaccharides (RFOs) are a kind of D-galactose-containing polysaccharide widely present in higher plants. These RFOs include raffinose and stachyose, as well as other derivatives. They function as protective osmolytes, significantly enhancing plant tolerance to abiotic stress (Liu et al., 2024).

The biosynthesis of RFOs is catalyzed by two core enzymes: galactinol synthase (GolS) and raffinose synthase (RS) (Sengupta et al., 2015). GolS catalyzes the condensation of uridine diphosphate-galactose (UDP-Gal) with myo-inositol to form galactinol. RS then catalyzes the transfer of the galactosyl moiety from galactinol to sucrose, producing raffinose. Subsequent stachyose formation is catalyzed by stachyose synthase (STS). STS utilizes raffinose and galactinol as substrates to transfer the galactosyl moiety from galactinol to the C6 position of the galactose unit in raffinose (Liu et al., 2024).

As the key enzymes catalyzing RFO biosynthesis, raffinose synthase family have been systematically identified and functionally characterized across diverse plants, including *Arabidopsis* (*Arabidopsis thaliana*) (Zuther et al., 2004; Peters et al., 2010; Egert et al., 2013), wheat (*Triticum aestivum*) (Guo et al., 2023), maize (*Zea mays*) (Zhou et al., 2012), rice (*Oryza sativa*) (Zhang et al., 2024), cotton (*Gossypium*) (Cui et al., 2021), soybean (*Glycine max*) (De Koning et al., 2021), cucumber (*Cucumis sativus*) (Sui et al., 2012), and sesame (*Sesamum indicum*) (You et al., 2018). Convincing evidence highlights the diverse functions of *RS* genes in stress adaptation and seed development, including seed desiccation tolerance and longevity (Liu et al., 2024). In *Arabidopsis*, six putative *RS* genes (*AtRS1–6*) have been identified (Nishizawa et al., 2008). Among these, AtRS2 protein displays an α-galactosidase activity but no evidence supporting RS activity (Peters et al., 2010). AtRS4 and AtRS5 are functionally confirmed as raffinose synthase, while *AtRS3* is a pseudogene (Egert et al., 2013). *AtRS5* is indispensable for raffinose biosynthesis in leaves, as its loss-of-function mutation abolishes leaf raffinose accumulation (Egert et al., 2013). AtRS4 functions primarily as a stachyose synthase in seeds, with its disruption leading to the complete absence of stachyose. Besides, AtRS4 possible also serve as the seed-specific raffinose synthase that contributes to raffinose accumulation under abiotic stress (Gangl et al., 2015), indicating that AtRS4 is a multifunctional enzyme with a key regulatory role in RFO biosynthesis. The *AtRS4/5* double mutant exhibits drastically reduced RFO levels and impaired seed germination under dark conditions, highlighting the functional redundancy and developmental specificity within the RS family (Gangl and Tenhaken, 2016). Similarly, in soybean (*Glycine max*), multiple *GmRS* genes have been identified, some of which exhibit drought-inducible expression (De Koning et al., 2021). In maize, both mRNA and protein of *ZmRS* are inducibly upregulated under drought stress, and raffinose accumulation correlates positively with drought resilience (Zhao et al., 2020). Two independent transposon-interrupted *zmrs* mutant lines display undetectable RS protein, accompanied by hyperaccumulating galactinol, absence of raffinose, and increased sensitivity to drought, and enhanced sensitivity to drought stress (Li et al., 2017, 2020). Overexpression of *ZmRS* in maize markedly increases leaf raffinose accumulation, reduces transpiration, and confers robust drought tolerance without compromising growth or grain yield (Liu et al., 2023). Intriguingly, heterologous expression of *ZmRS* in *Arabidopsis* enhanced plant drought tolerance despite a paradoxical decrease in leaf raffinose content, suggesting context-dependent regulatory mechanisms (Li et al., 2020). In wheat, *RS* homologs are upregulated during cold acclimation, contributing to membrane stabilization; Overexpression of *TaRS15-3B* in transgenic lines confers enhanced tolerance to both drought and salinity (Guo et al., 2023). Beyond stress mitigation, raffinose synthase is essential for seed desiccation tolerance and longevity during storage, underscoring its multifaceted biological functions (Salvi et al., 2022).

Barley (*Hordeum vulgare* L.), the fourth most important cereal crop worldwide, is recognized for its adaptability to marginal environments and its crucial roles in animal feed, malting, and human nutrition. As a diploid species, barley possesses seven chromosome pairs with a haploid genome size of approximately 4.8 Gb (Mascher et al., 2024). Notably, raffinose is widely distributed in plant kingdom and contributes to stress tolerance and seed development. Nevertheless, the key *RS* genes responsible for raffinose synthesis in barley remain unexplored. To fill the knowledge gap concerning the *RS* gene family, we performed a genome-wide identification of *HvRS* genes using bioinformatic approaches. The phylogenetic relationships, conserved domains, synteny, promoter cis-element, and expression profiles under abiotic stress conditions were analyzed. This comprehensive characterization of the *RS* gene family in barley establishes a solid foundation for future gene function analysis and genetic improvement of barley.

## 2 Materials and methods

### 2.1 Identification and physicochemical property prediction of *HvRS* gene family

To identify *RS* homologs in barley, a BLASTP search was conducted against the barley protein database (https://galaxy-web.ipk-gatersleben.de/) using known RS sequences from *Arabidopsis thaliana* (Nishizawa et al., 2008), rice (Zhang et al., 2024), and maize (Zhou et al., 2012) as queries, with an E-value threshold < 1E-20. The characteristic Pfam domain (PF05691) was retrieved from the Pfam database (https://pfam.xfam.org/). The Pfam domain HMM profile was further employed as a query to search against the Morex V3 barley proteins (https://doi.org/10.5447/ipk/2021/3) using HMMER 3.0 (Finn et al., 2011) with an E-value cutoff of 0.005. Proteins that satisfy the criteria of both the BLASTP and HMMER searches are considered as candidate HvRS proteins.

Protein properties, including amino acid number, molecular weight (Da), isoelectric point (pI), and grand average of hydropathicity (GRAVY), were computed with ExPASy ProtParam (https://web.expasy.org/protparam/). Subcellular localization was predicted using the online Plant-mPLoc server (http://www.csbio.sjtu.edu.cn/bioinf/plant-multi) (Chou and Shen, 2010).

### 2.2 Structure and conserved motif analysis of *HvRS* gene family

Conserved motifs of each HvRS protein were analyzed using the MEME Suite (http://meme-suite.org/tools/meme). Conserved domains of HvRS proteins were identified by NCBI Batch CD-search (https://www.ncbi.nlm.nih.gov/Structure/cdd/wrpsb.cgi). The conserved motifs, conserved domain, and gene structure were visualized using TBtools (Chen et al., 2023).

### 2.3 Phylogenetic analysis of HvRS

To investigate the phylogenetic relationships of HvRS proteins with their orthologs, 79 full- length RS protein sequences were selected from *Triticum aestivum*, *Zea mays*, *Oryza sativa*, *Brachypodium distachyon*, *Aarabidopsis thaliana*, *Setaria italica*, *Sorghum bicolor*, and *Hordeum vulgare*. These proteins were available in the papers (Guo et al., 2023; Xu et al., 2023) and were listed in **Supplementary Table 1**. Multiple sequence alignment was performed using ClustalW in MEGA7 (Kumar et al., 2016), and a phylogenetic tree was constructed with the maximum likelihood (ML) method. The resulting tree was visualized and refined using the online tool iTOL (https://itol.embl.de/).

### 2.4 Analysis of cis-acting elements in *HvRS* promoters

For each *HvRS*, the 2000-bp sequence upstream of the initiation codon (ATG) was extracted from the Morex V3 reference genome (Mascher et al., 2021) using TBtools (Chen et al., 2023).The cis-acting elements were predicted using the online software PlantCARE (http://bioinformatics.psb.ugent.be/webtools/plantcare/html/). The results were visualized with TBtools.

### 2.5 Expression profiling of *HvRS* genes

#### 2.5.1 Tissue-specific expression analysis of *HvRS* genes

The FPKM values (fragments per kilobase of transcript per million mapped reads) for Morex V3 transcriptome data from 16 tissues/stages (PRJEB14349) were retrieved from the BarleyExpDB database (Li et al., 2023). In this database, *HRS4* and *HvRS6* were not differentiated into *HvRS4.1* and *HvRS4.2* or *HvRS6.1* and *HvRS6.2*, both contained the expression levels of the two transcript variants. The heatmap was generated using the pheatmap package in R version 4.2.2, with rows scaled by Z-score normalization (Kolde and Kolde, 2015).

#### 2.5.2 qRT-PCR analysis of *HvRS* genes under stress treatment

A stress-tolerant barley landrace (ZDM01411) was obtained from the National Crop Genebank of China at the Institute of Crop Sciences, Chinese Academy of Agricultural Sciences. This accession, originating from Inner Mongolia, China, exhibits superior salt tolerance and moderate drought tolerance. Seeds were surface-sterilized with 3% sodium hypochlorite and 75% ethanol, then germinated on sterile filter paper, and grown for two weeks in a modified Hoagland nutrient solution (NSP1020, Coolaber, China) in a greenhouse under a 14-h light/10-h dark photoperiod at 22 °C. Seedlings were then subjected to nutrient solution with 200 mmol /L NaCl (Sinopbarm Chemical Reagent, Co., Ltd., China), 20% (w/v) PEG6000 (Solarbio, China), and 100 μmol/L abscisic acid (ABA) (Solarbio, China) for stress treatments respectively (Chang et al., 2024; Gao et al., 2024), while seedlings maintained in normal nutrient solution served as controls. Seedlings were harvested at 1 h, 6 h, 12 h, and 24 h post-treatment with three biological replicates, immediately frozen in liquid nitrogen, and stored at −80 °C until use.

Total RNA from the roots or shoots was extracted using the EASYspin Plus Plant RNA Extraction Kit (Aidlab, China). RNA quality was assessed using a Nanodrop One spectrophotometer (Thermo Fisher Scientific, USA) and agarose gel electrophoresis. First-strand cDNA was synthesized by the PrimeScript RT reagent kit with gDNA Eraser (TaKaRa, Japan). The qRT-PCR was performed using the SYBR Green Premix Pro TaqHS qPCR Kit (Accurate Biology, China) on QuantStudio 5 real-time PCR system (Applied Biosystems, USA). The *HvActin* gene (*HORVU.MOREX.r3.1HG0003140.1*) was used as an endogenous reference for normalization. The qRT-PCR assay was conducted in a 20 µL reaction volume comprising 10 µL of 2× SYBR Green *Pro* Taq HS Premix, 0.8 µL of primer mixture (10 µM each forward and reverse), 0.4 µL of ROX Reference Dye (4 µM), 2 µL of cDNA, and 6.8 µL of nuclease-free water. Thermal cycling was performed under the following conditions: initial denaturation at 95 °C for 30 s; 40 cycles of 95 °C for 5 s and 60 °C for 30 s. Melting curve analysis was subsequently carried out at 95 °C for 15 s, 60 °C for 1min, and 95 °C for 1 s. All samples were analyzed in triplicate technical replicates. The relative expression levels of the *HvRS* gene family were calculated using the 2^−ΔΔCt^ method (Livak and Schmittgen, 2001). The expression levels in the control of root sample at 1h were defined as “1”. Expression levels were directly compared between three distinct stress conditions and the control, specifically within the same tissue (roots or shoots) and at identical time points post-treatment. Data were analyzed by one-way ANOVA, followed by Tukey’s tesy for multiple comparisons test using GraphPad Prism 9 (GraphPad Software, Boston, MA, USA). Significance levels are denoted as * (*P* ≤ 0.05), **(*P* ≤ 0.01), *** (*P* ≤ 0.001). The primers used are listed in the **Supplementary Table 2**.

### 2.6 Haplotype analysis of *HvRS2* and *HvRS5*

Based on geographic origin, barley is classified into Western and Eastern types. Western barley, which predominantly comprises the two-row variety, originated from the Fertile Crescent, Europe, and North Africa. The Eastern type refers to barley originating from the Zagros region, extending across Asia to North Korea, and is primarily characterized by the six-row variety (Morrell and Clegg, 2007). The genetic diversity of *HvRS2* and *HvRS5* was analyzed using a comprehensive pan-genome from 76 barley varieties (Jayakodi et al., 2024). The details of the 76 barley varieties, including their status, country of origin, annuality, row type, and *HvRS2*/*HvRS5* haplotypes, are provided in **Supplementary Table 3**. The DNA sequences were aligned using the DNAMAN version 7 (Lynnon BioSoft, Canada). Haplotype analysis was conducted by DnaSP 6.0 (Rozas et al., 2017). A gene haplotype is a combination of alleles or genetic variants (such as SNPs, InDels) located at physically linked loci within a single gene on the same chromosome that are inherited together as a single unit (Hoehe, 2003). A haplotype network was generated in Popart v1.7 using the TCS network methods (Leigh et al., 2015).

## 3 Results

### 3.1 Identification of *RS* gene family in barley

A total of eight *RS* genes were identified in the barley cv. Morex V3 reference genome. These *HvRS* genes are distributed across four barley chromosomes: 1H, 2H, 3H, and 7H. Among them, *HORVU.MOREX.r3.1HG0046150* and *HORVU.MOREX.r3.7HG0656670* each contain two transcripts, designated *HvRS4.1*, *HvRS4.2* and *HvRS6.1*, *HvRS6.2*, respectively (**Table 1**). Gene functional annotation indicated that all *RS* genes were implicated in raffinose biosynthesis. HvRS1, HvRS2, and HvRS3 were annotated as alkaline alpha-galactosidase seed imbibition proteins, HvRS4.1 and HvRS4.2 were alpha-galactosidases. HvRS5, HvRS6.1, and HvRS6.2 were raffinose synthase family proteins.

**Table 1:** Characteristics of the *RS* genes in barley and their protein physicochemical properties.

Gene name	Gene ID	Chromosome location	Exon number	ORF (bp)	AA (aa)	Mw (Da)	PI	GRAVY	Sub-cellular location	Description
HvRS1	HORVU.MOREX.r3.7HG0677770.1	chr7H:159606996-159611350	13	2247	749	81347.79	5.92	-0.192	Cytoplasm.	Alkaline alpha-galactosidase seed imbibition protein
HvRS2	HORVU.MOREX.r3.3HG0237880.1	chr3H:54662734-54666325	8	2271	757	82133.74	5.7	-0.192	Chloroplast, Cytoplasm, Mitochondrion.	Alkaline alpha-galactosidase seed imbibition protei
HvRS3	HORVU.MOREX.r3.2HG0176000.1	chr2H:530097809-530101867	14	2421	807	87831.79	5.67	-0.12	Chloroplast, Cytoplasm.	Alkaline alpha-galactosidase seed imbibition protein
HvRS4.1	HORVU.MOREX.r3.1HG0046150.1	chr1H:303581211-303586895	15	1209	403	44014.29	7.04	-0.441	Cell wall.	Alpha-galactosidase
HvRS4.2	HORVU.MOREX.r3.1HG0046150.2	chr1H:303581211-303586895	14	966	322	35338.66	5.88	-0.424	Cell wall, Chloroplast.	Alpha-galactosidase
HvRS5	HORVU.MOREX.r3.3HG0233820.1	chr3H:32881770-32884866	2	2346	782	85121.88	5.48	-0.078	Chloroplast, Cytoplasm.	Raffinose synthase family protein
HvRS6.1	HORVU.MOREX.r3.7HG0656670.1	chr7H:50647116-50650000	4	2301	767	81485.36	5.54	-0.023	Chloroplast, Cytoplasm.	Raffinose synthase family protein
HvRS6.2	HORVU.MOREX.r3.7HG0656670.2	chr7H:50647116-50650284	5	2562	854	90465.54	6.09	-0.074	Chloroplast.	Raffinose synthase family protein

GRAVY: Grand average of hydropathicity.

The HvRS proteins ranged from 322 (HvRS4.2) to 854 (HvRS6.2) amino acids in length, and their predicted molecular weights between 35.3 kDa and 90.5 kDa. Except for HvRS4.1, all proteins were acidic, exhibiting PI values below 6.5. The GRAVY values (ranging from –0.441 to –0.023) indicated that all members of HvRS family are hydrophilic. Subcellular localization predictions suggested that seven RS proteins were located to the chloroplast and/or cytoplasm, while HvRS4.1 was exclusively localized in the cell wall.

### 3.2 Phylogenetic and collinearity analysis of RS protein family

**Figure 1:**
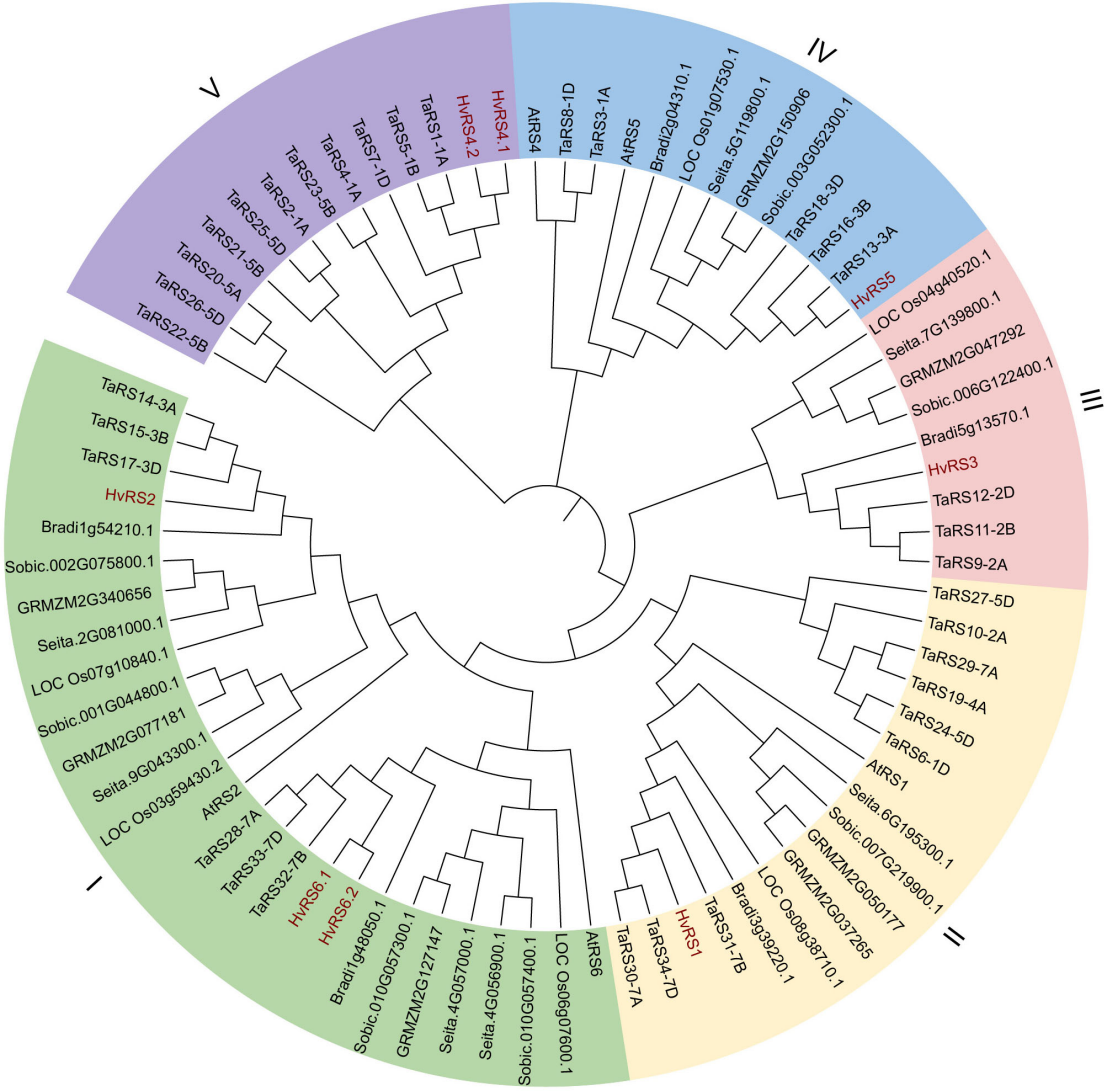
The phylogenetic trees of RS proteins constructed using the maximum likelihood (ML) method. The 79 RS proteins were derived from *Triticum aestivum*, *Zea mays*, *Oryza sativa*, *Brachypodium distachyon*, *Aarabidopsis thaliana*, *Setaria italica*, *Sorghum bicolor*, and *Hordeum vulgare*.

Phylogenetic analysis showed that 79 RS proteins from *Arabidopsis thaliana*, *Oryza sativa*, *Zea mays*, *Triticum aestivum*, *Brachypodium distachyon*, *Setaria italica*, *Sorghum bicolor*, and *Hordeum vulgare* were categorized into five distinct clades (**Figure 1**). The largest group, Clade I, comprised 27 members, including HvRS2, HvRS6.1, and HvRS6.2. Clade II contained 17 members, including HvRS1. Furthermore, HvRS3, HvRS5, and HvRS4.1-4.2 were assigned to Clades III, IV, and V, respectively. The RS proteins from barley, wheat, and *Brachypodium distachyon* were closely clustered, highlighting their strong evolutionary conservation.

**Figure 2:**
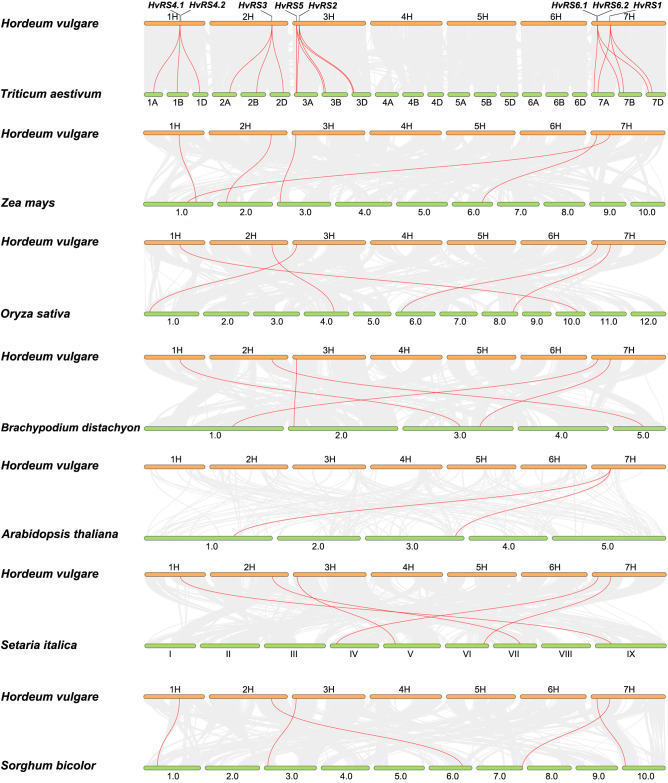
Comparative collinearity analysis of the *RS* gene orthologs among eight plants. Collinear *RS* gene pairs are highlighted in red.

Inter-species collinearity analysis revealed a large number of conserved syntenic blocks between barley and seven other representative plant species (**Figure 2**). Notably, six *HvRS* genes exhibited 18 collinear gene pairs with orthologs in *Triticum aestivum*. Furthermore, five *RS* gene pairs were identified between barley and *Zea mays*, *Oryza sativa*, *Brachypodium distachyon*, *Setaria italica*, and *Sorghum bicolor*. These results suggest that the majority of *RS* genes have been evolutionarily conserved across species, underscoring their important and conserved biological functions.

### 3.3 Conserved motifs, domain, and gene structure analysis

**Figure 3:**
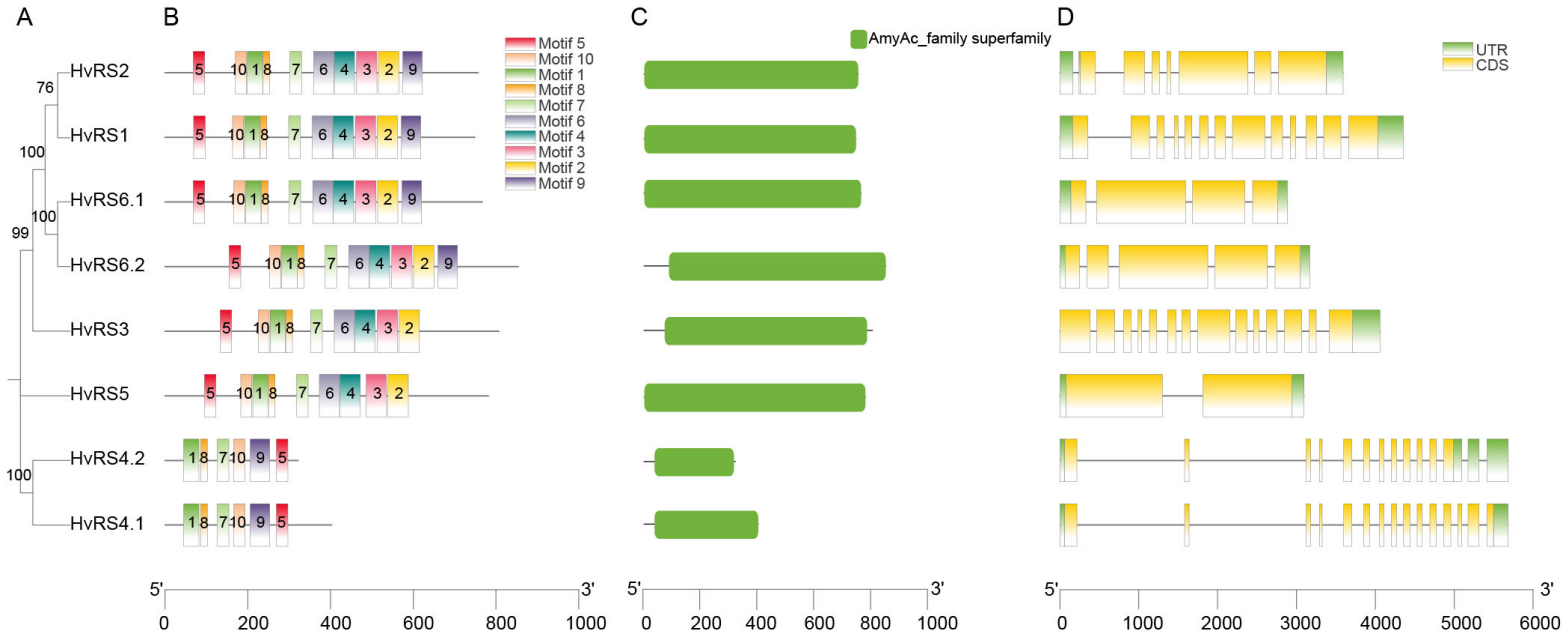
Conserved motifs, conserved domains, and gene structures of *HvRS* genes. **(A)** Phylogenetic tree of HvRS subfamilies. **(B)** Distribution of conserved RS motifs. **(C)** Conserved protein domain belonging to AmyAC family superfamily. **(D)** Exon-intron structures of *HvRS* genes.

The MEME-predicted protein motif analysis revealed striking similarity in motif types and numbers among HvRS proteins, except for HvRS4.1 and HvRS4.2 (**Figure 3B**). Specifically, HvRS1, HvRS2, HvRS6.1, and HvRS6.2 shared all ten predicted motifs. HvRS3 and HvRS5 lacked only motif 9. While HvRS4.1 and HvRS4.2 contained only six motifs (1, 5, 7, 8, 9, and 10). These motif distribution patterns are consistent with the phylogenetic relationships (**Figure 3A**). Furthermore, all HvRS family members contain the conserved domain (AmyAc-family super family) (**Figure 3C**). Analysis of exon-intron structures revealed considerable variation in exon numbers among *HvRS* genes, ranging from 2 to 15 (**Figure 3D**). The presence of both conserved motifs and variable structural features within the *RS* gene family indicates functional divergence over the course of evolution, suggesting that they may fulfill distinct biological functions.

### 3.4 Cis-acting element analysis of *HvRS* gene promoter regions

**Figure 4:**
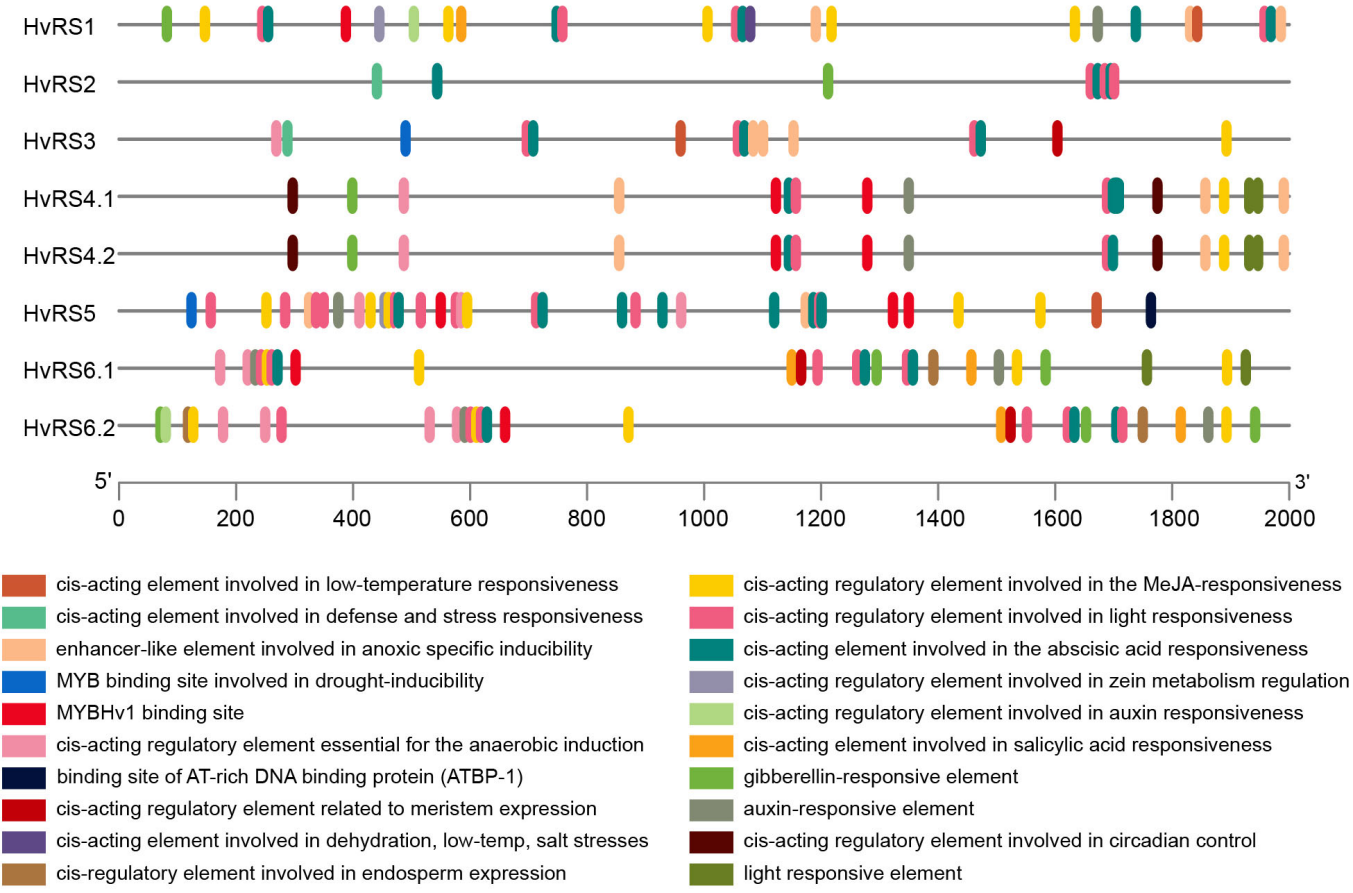
Cis-acting elements in promoters of *HvRS* genes.

The number of cis-acting elements identified in these promoters ranged from 8 to 29 (**Figure 4** and **Supplementary Table 4**). Hormone-related elements, specifically those responsive to ABA, methyl jasmonate (MeJA), and auxin, were most abundant, accounting for 42.7% of all elements, followed by light-responsive (24.3%) and stress-responsive (19.7%) elements. The elements associated with growth and development, and MYB-binding sites accounted for only 0.08% and 0.06%, respectively. The presence of cis-acting elements related to drought induction and salt stress suggests that the *HvRS* gene family may play a significant role in abiotic stress response.

### 3.5 Expression pattern analysis of *HvRS* genes

**Figure 5:**
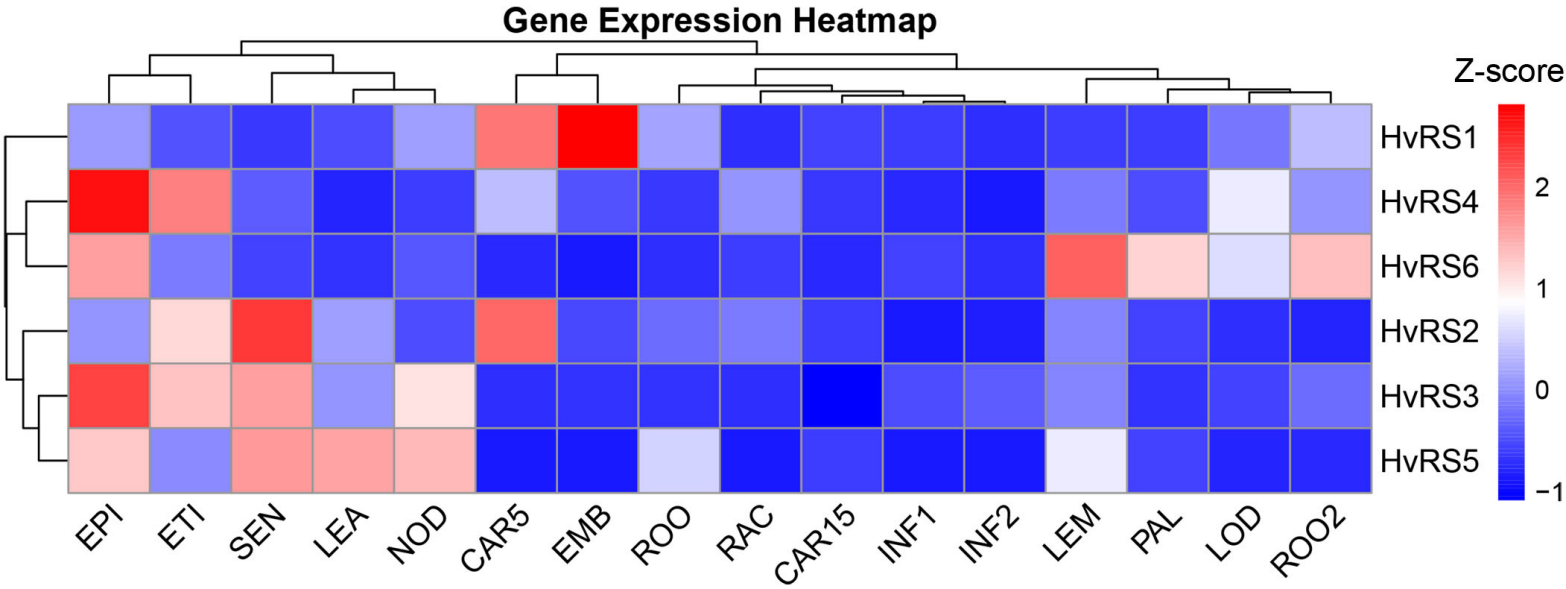
Relative expression of *HvRS* genes in different tissues of barley. CAR15: Grain with bracts removed at 15 DPA; CAR5: Grain with bracts removed at 5 DPA; EMB: Embryos dissected from 4d germinating grains; EPI: Epidermis (4 weeks); ETI: Etiolated from 10 day old seedling; INF1: Young Inflorescences (5 mm); INF2: Inflorescences (1-1.5 cm); LEA: Shoot from the seedlings (10 cm shoot stage); LEM: Lemma (6 weeks after anthesis); LOD: Lodicule (6 weeks after anthesis); NOD: Developing tillers at six leaf stage; PAL: Palea (6 weeks after anthesis); RAC: Rachis (5 weeks after anthesis), ROO: Root from 4 weeks seedlings; ROO2: Roots from the seedlings at 10 cm shoot stage; SEN: Senescing leaf (2 months).

We utilized publicly available transcriptome data to analyze the tissue expression differences among *HvRS* genes. The *HvRS* gene members displayed distinct tissue-specific expression patterns based the FPKM values (**Figure 5**). *HvRS4* showed high expression (FPKM > 50) in nearly every tissue, particularly in EPI (Epidermis, 4 weeks), ETI (Etiolated, 10 day old seedling), and LOD (Lodicule, 6 weeks PA). *HvRS2* showed high expression in SEN (Senescing leaf, 2 months), CAR5 (Grain, bracts removed, 5 DPA), and ETI (Etiolated, 10 day old seedling), the expressions in INF1 (Young Inflorescences, 5 mm) and INF2 (Inflorescences, 1-1.5 cm) were nearly absent (FPKM value ≤3). *HvRS3* was widespread expression with high express in EPI, SEN, ETI, and NOD (Developing tillers, six leaf stage). *HvRS1* showed obvious tissue-preferential expression, with high expression in EMB (Embryos, 4d dissected from germinating grains) and CAR5, and most other tissues showed low expression levels. *HvRS6* shows moderate expression (10 ≤ FPKM ≤ 50) in LEM (Lemma, 6 weeks PA), EPI, ROO2 (Roots from the seedlings, 10 cm shoot stage), PAL (Palea, 6 weeks PA), and LOD. *HvRS5* shows minimal expression across all tissues, mainly expression in LEA, NOD, EPI, LEM and ROO1 (Root, 4 weeks).

**Figure 6:**
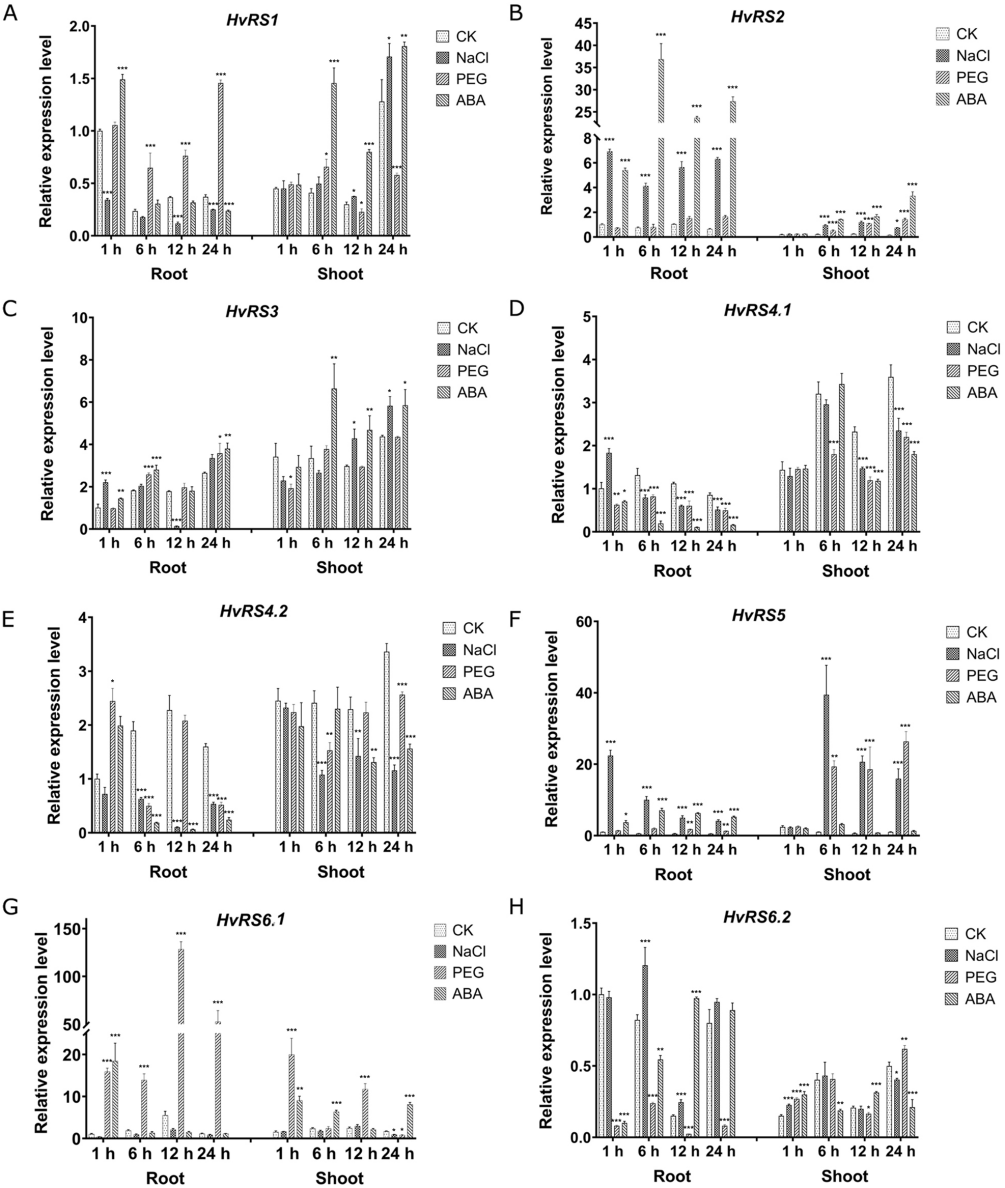
Relative expression levels of *HvRS* genes in the roots and shoots at the seedling stage under treatments of 200 mmol/L NaCl, 20% (w/v) PEG6000, and 100 μmol/L ABA. Data were assessed by one-way ANOVA with Tukey’s multiple comparisons test. Significance levels are denoted by asterisks: *(*P* ≤ 0.05), **(*P* ≤ 0.01), and ***(*P* ≤ 0.001).

To further evaluate the potential role of *HvRS* genes in response to abiotic stress, we performed gene expression analysis via qRT-PCR under three abiotic stress conditions: salinity, PEG-induced drought, and ABA treatment (**Figure 6**). After 6 h of treatment, barley seedlings exposed to PEG and NaCl displayed clear symptoms of leaf dehydration and wilting, whereas those treated with ABA showed no obvious phenotypic changes. Notably, the expression of *HvRS2* in roots was significantly upregulated by both salt and ABA treatments. Specifically, compared with time-matched controls, the expression levels in roots under salt stress were 6.91-fold (1 h), 5.49-fold (6 h), 5.45-fold (12 h), and 9.90-fold (24 h); under ABA treatment, they were 5.39-fold (1 h), 49.1-fold (6 h), 22.9-fold (12 h), and 43.0-fold (24 h). Additionally, the expression of *HvRS2* in shoots remained at a low and stable level during the initial stage of treatment (1 h) but increased significantly at 6 h, 12 h, and 24 h under all three abiotic stress conditions.

In root, *HvRS5* exhibited a similar expression pattern to *HvRS2*, being up-regulated by salt stress (22.4-, 17.5-, 9.56-, and 8.11-fold at 1, 6, 12, and 24 h) and ABA (3.68-, 12.4-, 12.1-, and 10.3-fold at the corresponding time points). In shoots, the *HvRS5* expressions were highly up-regulated after 6 h, 12 h, and 24 h under salinity and PEG treatment, reaching fold-change values of 40.0, 34.9, and 16.1 under salt stress, and 19.6, 31.3, and 26.6 under PEG stress. Although *HvRS6.1* exhibited an upward expression trend under stress conditions, its overall expression level remained still relatively low, as evidenced by based on the high ΔCt values relative to the internal reference gene. It is noteworthy that the expression level of the *HvRS4.1* gene in the root was reduced at 6 h, 12 h, and 24 h after ABA treatment. The results suggest that the *HvRS2* and *HvRS5* genes are likely involved in regulating abiotic stress responses during the early growth stages.

### 3.6 Pan-genome haplotype analysis of *HvRS2* and *HvRS5*

**Figure 7:**
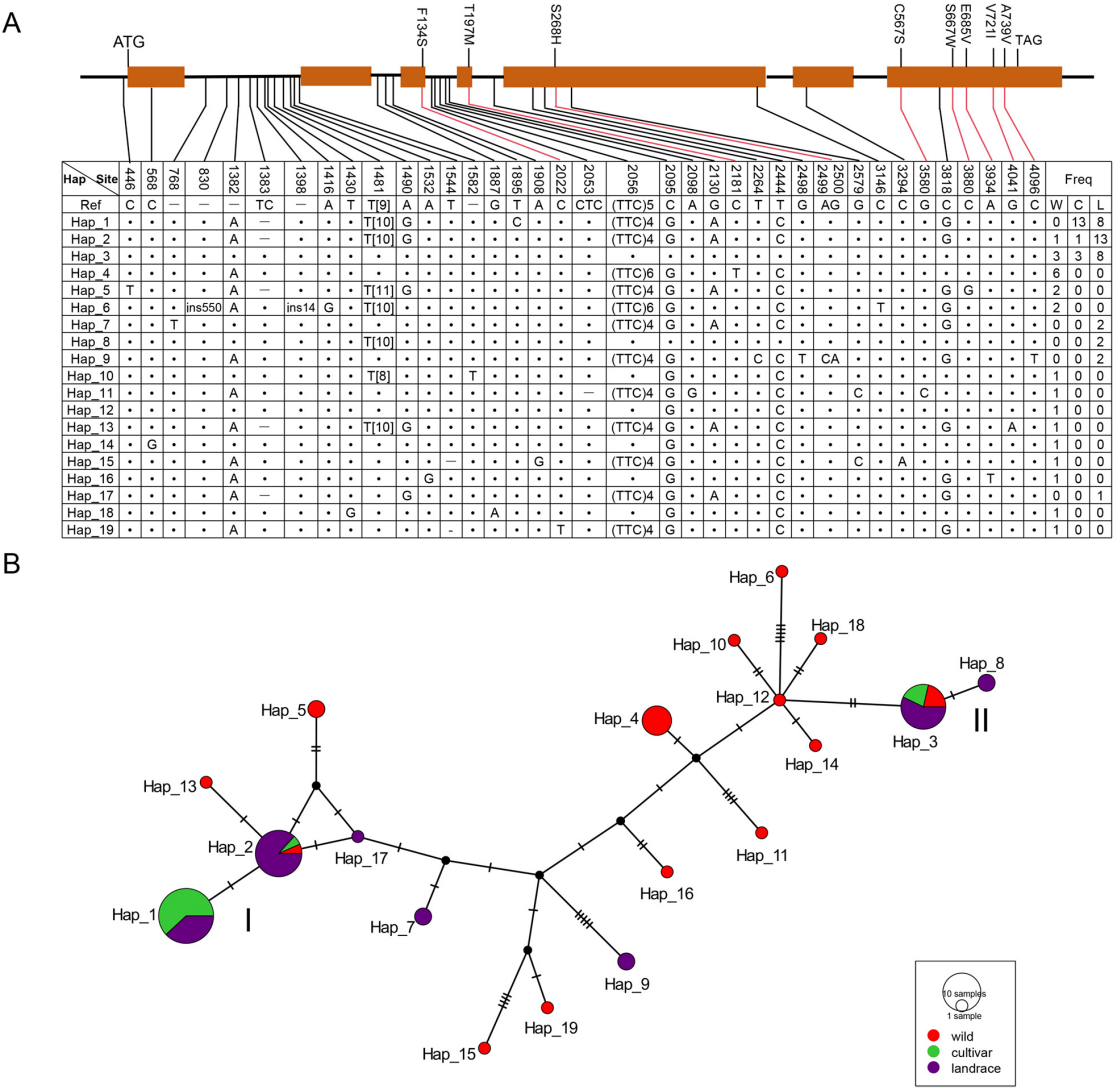
Allelic diversity and haplotype network analysis of *HvRS2.***(A)** Haplotype analysis of *HvRS2* around pan-genome from 76 barleys. Solid dark brown rectangles represent exons. The abbreviations W, C, and L represent wild, cultivated, and landrace barley respectively. “Freq” is the abbreviation of “frequency”, indicating the number of different types of barley accessions. The dot symbol indicates consistency with the reference sequence. A hyphen (−) denotes missing. The “ins550” and “ins14” indicate 550-bp and 14-bp insertions, respectively. “T[8]”, “T[9]”, and “T[10]” indicate 8, 9, and 10 thymine repeat. **(B)** Haplotype network analysis of *HvRS2*. Size of the pie chart is proportional to the haplotype frequency. Mutations between haplotypes are shown as lines representing mutations from the common haplotype.

To investigate the environmental adaptation and evolutionary patterns of *HvRS* genes, we selected the two stress upregulated genes (*HvRS2* and *HvRS5*) to conduct allelic variation analysis based on pan-genome data from 76 barley genotypes. Sequence analysis showed that the coding sequence of *HvRS2* was highly conserved in domesticated barley (including cultivars and landraces) with no amino acid variation, despite numerous polymorphic sites in wild barley. A total of 19 haplotypes were identified for *HvRS2*. The wild accessions contained 14 haplotypes, whereas cultivars and landraces harbored 3 and 7, respectively (**Figure 7A**). The haplotypes of domesticated barley clustered into two main groups: Group I (Hap_1, Hap_2, Hap_7, Hap_17) and Group II (Hap_3, Hap_8), revealing clear genetic divergence between Eastern and Western barley (**Figure 7B**). Group I accessions were primarily Western barley originating from Turkey, Syria, Iran, and Ethiopia. In contrast, Group II accessions were Eastern barley, largely derived from East and Central Asia, such as China, Pakistan, Saudi Arabia, Afghanistan, and Russia.

Compared to *HvRS2*, the *HvRS5* gene exhibited more SNPs and amino acid variations. A total of 25 haplotypes were identified in *HvRS5*. The wild, landraces, and cultivars owed 21, 9, and 4 haplotypes, respectively. In domesticated barley, Hap_1, Hap_2, Hap_3, and Hap_4 were predominant, accounting for 88.7% (**Supplementary Figure 1A**). These haplotypes were categorized into three distinct groups: Group I (Hap_1 and Hap_3), Group II (Hap_2 and Hap_20), and Group III (Hap_4). Similarly, a clear genetic divergence of *HvRS5* was also observed between Eastern and Western barley (**Supplementary Figure 1B**). Group I haplotypes were mainly Western barley, whereas Group II haplotypes were primarily the Eastern barley. Group III contained both Eastern barley and Western barley. We speculated that the distinct haplotypes of the *HvRS2* and *HvRS5* genes may have undergone domestication selection at different geographic locations.

## 4 Discussion

Raffinose synthase is crucial in plant physiology as it catalyzes the synthesis of raffinose from galactinol and sucrose, driving carbohydrate metabolism for carbon partitioning and storage (Yan et al., 2022; Liu et al., 2024). Raffinose and its derivatives function as osmolytes or as signaling molecules, contributing to enhanced plant tolerance against a range of abiotic stresses. Additionally, they are involved in phloem transport, antioxidant activity, seed desiccation tolerance, longevity, and germination vigor (Nishizawa-Yokoi et al., 2008). Thus, *RS* genes are primary targets for improving crop resilience and yield stability under changing climates. However, the *RS* gene family in barley has not been fully characterized.

In this study, we systematically identified eight *HvRS* genes and explored their phylogenetic relationships, structural features, promoter cis-acting elements, expression patterns, and natural allelic variation. The phylogenetic analysis, clustering HvRS proteins into five distinct clades with orthologs from other grasses, underscores a deep evolutionary conservation of this gene family. The particularly close relationship of RS proteins between barley, wheat, and *Brachypodium distachyon* reflects they share a conserved evolutionary pathway for RFO biosynthesis. This deduction is further robustly supported by the interspecies collinearity analysis, which revealed numerous syntenic blocks and gene pairs among gramineous crops, especially with triticeae. The analysis of conserved motifs, domains, and gene structures offers clues to the functional divergence within the *HvRS* family. HvRS4.1 and HvRS4.2, possessing only six motifs and forming a separate clade (V), likely underwent significant functional specialization. The presence of genes with *HvRS4* and *HvRS6* transcripts hints at potential regulatory complexity via alternative splicing, a mechanism that can increase functional diversity from a limited gene repertoire (Alhabsi et al., 2025). We infer that *HvRS4* and *HvRS6* transcripts are likely expressed in specific tissues or at particular developmental stages. Such spatiotemporal expression patterns would allow them to participate in raffinose synthesis in a more precisely regulated manner, thereby playing specialized roles in biological processes such as seed development or stress response (Xu et al., 2023). More rigorous gene expression experiments will be required to further validate this hypothesis. The diversity in exon number (2 to 15) further indicates evolutionary divergence, potentially influencing transcript stability, alternative splicing patterns, and regulatory responsiveness, thereby fine-tuning the functions of different family members. The preservation of these genes over millions of years of evolution strongly implies indispensable, conserved biological functions, such as stress tolerance and carbon metabolism.

The cis-acting element analysis of promoter regions reveals a regulatory landscape that is strongly biased towards environmental and hormonal signals (Hernandez-Garcia and Finer, 2014). The notable abundance of hormone-responsive elements (ABA, MeJA, and auxin) and stress-responsive elements is well aligned with the established role of RFOs in abiotic stress adaptation. These promoter cis-acting elements may regulate the expression of *HvRS* genes, especially in response to drought, salinity, and associated hormonal signals such as ABA. Stress-responsive and tissue-specific and gene expression are crucial for further understanding gene function. For the stress-responsive gene expression analysis, the upregulation of *HvRS2* and *HvRS5* in roots and shoots under salt, PEG-simulated drought, and ABA treatments identifies them as key stress-responsive genes. Their rapid induction suggests a role in early osmotic adjustment and cellular protection (ElSayed et al., 2014). The observed downregulation of *HvRS4.1* in roots upon ABA treatment presents a noteworthy finding, which is likely to be a negative regulatory gene in response to adverse conditions. Further in-depth studies, including knockout, overexpression, and other molecular biology experiments, are required to elucidate the underlying regulatory mechanism. RS has been shown to play a vital role in abiotic stress tolerance in various plant species. Under drought, salinity, cold, and oxidative stress, plants such as *Arabidopsis* (Egert et al., 2013), wheat (Guo et al., 2023), and maize (Liu et al., 2023) exhibit significant accumulation of raffinose and elevated expression of *RS* genes. In line with this, *RS* knockout mutants show reduced raffinose levels and increased stress sensitivity. These results indicate that *RS* genes are multifunctional stress proteins involved with plant stress resistance.

The near-ubiquitous expression of *HvRS4* points to a fundamental, housekeeping role, possibly in maintaining basal levels of RFOs for cellular homeostasis or primary metabolism across tissues. Conversely, the strong, preferential expression of *HvRS1* in embryos and developing grains (CAR5, EMB) highlights a specialized function in seed development, desiccation tolerance, and seed vigor. The expression of *HvRS2* in senescing leaves (SEN) and grains, and *HvRS3* in epidermis and nodes, suggests their roles in source-sink carbon remobilization during senescence and in protective barriers or transport tissues, respectively (Schneider and Keller, 2009). The minimal expression of *HvRS5* under normal conditions contrasts sharply with its stress inducible pattern, marking it as a specialist gene activated primarily under adverse conditions.

Pan-genome haplotype analysis of the stress-responsive genes *HvRS2* and *HvRS5* provides critical insights into their population genetic structure and domestication trajectories. *HvRS2* exhibits strikingly high sequence conservation across domesticated barley accessions, in contrast to substantial polymorphism in wild relatives—a pattern strongly indicative of a selective sweep during domestication. Notably, the fixation of specific *HvRS2* haplotypes likely conferred adaptive advantages under cultivated conditions. In contrast, *HvRS5* displays greater haplotype diversity with more non-synonymous substitutions in domesticated barley. Both genes exhibit obvious geographic distribution of haplotypes, resolving into distinct Eastern and Western clades that align with established barley migration routes (Pankin et al., 2018; Civáň et al., 2021). Our study still has several limitations that warrant further research. The following work should include a broader range of barley genotypes, encompassing both tolerant and non-tolerant accessions, alongside a comprehensive analysis of stress physiology, raffinose and related metabolite profiles, and raffinose synthase (RS) activity. Future research will focus on identifying stress tolerance phenotypes in germplasm resources, and analyzing correlations between specific *HvRS* haplotypes and stress response traits. The key objectives include pinpointing the crucial variations of *HvRS* gene family accountable for stress-resistant phenotypes, as well as clarifying the corresponding molecular mechanisms through integrated multi-omics techniques and biotechnological methods. These efforts will provide essential genetic markers and candidate genes for molecular breeding programs aimed at developing stress-resistant cultivars. Collectively, this work offers a comprehensive characterization of the *HvRS* gene family in barley and lays a solid foundation for subsequent functional validation and genetic improvement.

## 5 Conclusion

In summary, we systematically identified eight *HvRS* genes and revealed their physicochemical property, evolutionary relationship, distinct regulatory and expression patterns. qRT-PCR analysis revealed *HvRS2* and *HvRS5* as key stress-responsive genes involved in abiotic stress tolerance. Furthermore, the haplotypes of these two genes in domesticated barley exhibited a clear East-West geographic divergence. Overall, this study establish a foundation for further functional analysis of *HvRS* genes and offer crucial insights into the molecular mechanisms underlying barley abiotic stress responses.

## Data Availability

The datasets presented in this study can be found in online repositories. The names of the repository/repositories and accession number(s) can be found in the article/**Supplementary Material**.

## Supplementary material

Supplementary Figure 1 Allelic diversity and haplotype network analysis of *HvRS5.***(A)** Haplotype analysis of *HvRS5* around pan-genome from 76 barley genotypes. Solid dark brown rectangles represent exons. The abbreviations W, C, and L represent wild, cultivated, and landrace barley. The dot (.) denotes consistency with the reference sequence; “−” represents the missing of the base, “delins7” and “delins75” indicate deletions of 7-bp and 75-bp. **(B)** Haplotype network analysis of *HvRS5.*

Supplementary Table 1 The 79 RS protein sequences employed in phylogenetic analysis.

Supplementary Table 2 The primer sequences of *HvRS* genes used for qRT-PCR analysis.

Supplementary Table 3 Details of the 76 barley genotypes used for allelic diversity and haplotype network analysis.

Supplementary Table 4 Detailed information on the cis-acting elements of the *HvRS* genes.
