# Supplementary figures and images for: Genome-wide identification of raffinose synthase gene family in barley (Hordeum vulgare L.) reveals its role in multiple abiotic stress tolerance
Source: Front Plant Sci. 2026 Mar 10;17:1767123. doi: 10.3389/fpls.2026.1767123 (PMC13008640; doi:10.3389/fpls.2026.1767123)

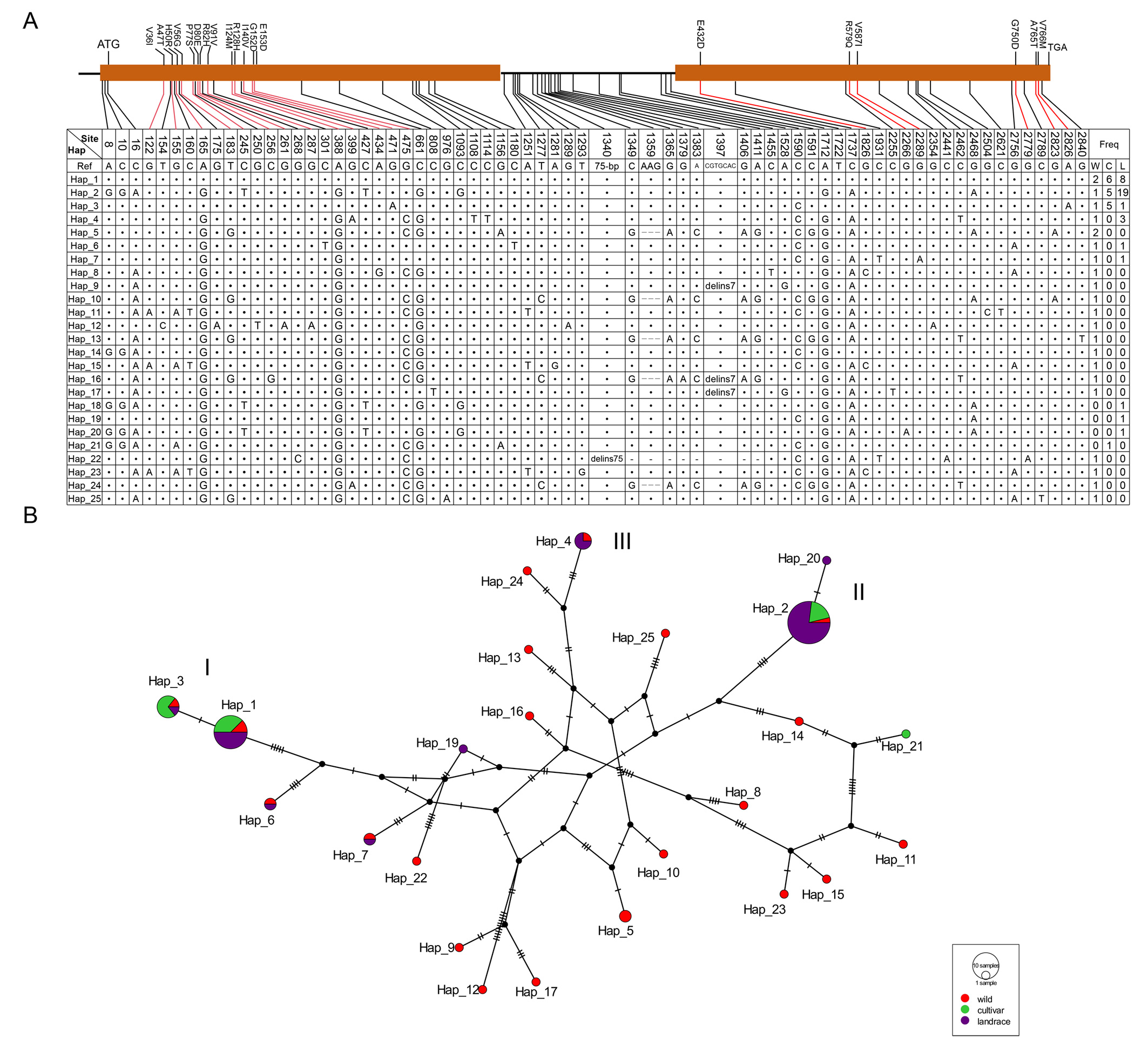

Supplement: Supplementary Figure 1 — Allelic diversity and haplotype network analysis of HvRS5.(A) Haplotype analysis of HvRS5 around pan-genome from 76 barley genotypes. Solid dark brown rectangles represent exons. The abbreviations W, C, and L represent wild, cultivated, and landrace barley. The dot (.) denotes consistency with the reference sequence; “−” represents the missing of the base, “delins7” and “delins75” indicate deletions of 7-bp and 75-bp. (B) Haplotype network analysis of HvRS5. [file Image1.jpeg]
